# 

**Published:** 1924-07

**Authors:** 


					CONTENTS.
Page
Tl
e Therapeutic Uses of Radium.
By A. E. Hayward Pinch.
*.R.C.S.
97
'Verticuijtis 0f the Pelvic Colon.
By Sir C. Gordon-Watson.
C.M.G., F.R.C.S.
ecovery after Mechanical Obstruction due to the Passage of
? Gallstone through the Intestine, with Saptic Peritonitis.
By
Charles A. Morton, F.R.C.S.
126
i labyrinthitis.
By E. Watson-Williams, M.C., Ch.M.
135
eviews of Books
PrJU- . .
I4i
_ d'torial Notes
148
^eetings of Societies
156
?bitu
ary
Edmund Comer Board
*57
Frederick George Farr
158
Forbes Fraser, C.B.E., F.R.C.S.
159
"fk .   ' '   ??? "? ??? ???
6 library of the Bristol Medico-Chirurgical Society
162
?Ca' Medical Notes
163

				

